# Salvage Treatment of Ventilator-Associated Pneumonia Using Sulbactam–Durlobactam in a Preterm Neonate: A Case Report

**DOI:** 10.3390/idr18030054

**Published:** 2026-06-01

**Authors:** Suzana Zivojinovic, Tijana Prodanovic, Nikola Prodanovic, Rasa Medovic, Milica Cekerevac, Dragana Savic, Bojana Markovic, Slobodan Jankovic

**Affiliations:** 1 Department of Pediatrics, Faculty of Medical Sciences, University of Kragujevac, 34000 Kragujevac, Serbia; zivojinovicsuzana@yahoo.com (S.Z.); rasamedovic@gmail.com (R.M.); milicacekerevac@gmail.com (M.C.); drsavicdragana@gmail.com (D.S.); bojana.kovacevic96@gmail.com (B.M.); 2 University Clinical Center Kragujevac, 34000 Kragujevac, Serbia; nikolaprodanovickg@gmail.com (N.P.); slobnera@gmail.com (S.J.); 3 Department of Surgery, Faculty of Medical Sciences, University of Kragujevac, 34000 Kragujevac, Serbia; 4 Department of Pharmacology and Toxicology, Faculty of Medical Sciences, University of Kragujevac, 34000 Kragujevac, Serbia

**Keywords:** ventilator-associated pneumonia, preterm neonate, *Acinetobacter baumannii*, sulbactam–durlobactam, ceftazidime–avibactam, multidrug resistance, neonatal intensive care unit

## Abstract

Background: Ventilator-associated pneumonia (VAP) is a leading cause of nosocomial infections in neonatal intensive care units (NICUs) and is associated with significant morbidity, mortality, and prolonged hospitalization, particularly in preterm neonates. Management is complicated by the emergence of multidrug-resistant (MDR) and extensively drug-resistant (XDR) pathogens, with very limited evidence supporting the use of reserve antibiotics in this population. Case Presentation: We report a male neonate born at 25 weeks of gestation (birth weight 740 g) who developed severe VAP caused by XDR *Acinetobacter calcoaceticus-baumannii* complex and *Pseudomonas aeruginosa*. After failure of multiple antibiotic regimens, including ampicillin, amikacin, meropenem, vancomycin, cefepime with inhaled colistin, tigecycline, and ceftazidime-avibactam combined with fosfomycin, the infant was treated with sulbactam–durlobactam (25 mg/kg/dose every 6 h) in combination with ceftazidime-avibactam. After 12 days of this regimen, the neonate was successfully extubated. Conclusions: This case highlights the therapeutic challenges of XDR infections in extremely preterm neonates and suggests that sulbactam–durlobactam may represent a viable salvage treatment option. Further pharmacokinetic and clinical studies are needed to establish optimal dosing and safety in the neonatal population.

## 1. Introduction

Ventilator-associated pneumonia (VAP) is a nosocomial pulmonary infection occurring in patients who require invasive mechanical ventilation for more than 48 h and who present without signs of pneumonia or incubating infection at the time of admission [1]. The incidence of nosocomial infections in neonatal intensive care units (NICUs) is approximately 30% in developing countries, with VAP being one of the leading causes of hospital-acquired infections in preterm neonates. This type of pneumonia significantly contributes to increased morbidity and mortality, requires prolonged hospitalization, and consequently increases healthcare costs [2].

Reported VAP incidence rates vary considerably, reflecting differences in gestational age, diagnostic criteria, and geographic location. In the United States, the incidence ranges from 0.3 to 1.6 cases per 1000 ventilator-days in NICUs [3]. Risk factors for nosocomial pneumonia in neonates and preterm infants include an underdeveloped immune system, sepsis, duration of mechanical ventilation, number of reintubations, use of broad-spectrum antibiotics, and administration of corticosteroids [1,4].

The most common causative organisms of VAP are Klebsiella pneumoniae, *Acinetobacter baumannii*, *coagulase-negative staphylococci*, and *Pseudomonas aeruginosa* [5,6,7]. Diagnosis of neonatal VAP is largely based on criteria defined by the Centers for Disease Control and Prevention (CDC) for infants younger than one year of age. According to CDC criteria, diagnosis requires at least 48 h of invasive mechanical ventilation, a positive chest radiograph (new or progressive and persistent infiltrate, consolidation, cavitation, or pneumatocele), impaired gas exchange, and the presence of at least three of the following: temperature instability, bradycardia or tachycardia, leukopenia or leukocytosis, respiratory distress, and appearance of mucopurulent secretions on tracheal aspiration. The tracheal aspirate culture is most commonly used to identify the causative organism; however, due to the difficulty in distinguishing true infection from colonization, results must always be correlated with clinical, laboratory, and radiographic findings [8,9].

Available data on the treatment of neonatal VAP largely relies on experience derived from the management of neonatal sepsis. In most centers, empirical antibiotic therapy for early-onset pulmonary infection typically includes ampicillin plus an aminoglycoside, with subsequent adjustment based on susceptibility testing once the causative organism is identified. For late-onset pneumonia, the choice of empirical therapy depends on local bacterial resistance patterns. Vancomycin and cephalosporins (cefotaxime) are commonly used to cover neonatal pathogens, including *coagulase-negative staphylococci* and *methicillin-resistant Staphylococcus aureus* (MRSA). When *Pseudomonas aeruginosa* infection is suspected, a combination of an aminoglycoside and an antipseudomonal beta-lactam, such as ceftazidime, cefepime, or piperacillin-tazobactam, is recommended. The recommended duration of therapy for uncomplicated pneumonia is 7 to 10 days. Inhaled antibiotic administration may reduce drug toxicity by limiting systemic absorption. Several studies have demonstrated a positive effect of combined intravenous and inhaled colistin in preterm neonates with VAP caused by *Acinetobacter baumannii* [10]. Prevention measures include ensuring a clean environment for intubated neonates (hand hygiene, sterile equipment handling), appropriate head-of-bed positioning to prevent gastric reflux, and daily reassessment of readiness for extubation [11].

We present a case of an extremely preterm neonate who developed VAP caused by extensively drug-resistant XDR *Acinetobacter calcoaceticus-baumannii* complex and *Pseudomonas aeruginosa*, who required escalating antibiotic therapy and was ultimately treated with off-label sulbactam–durlobactam.

## 2. Case Description

A male neonate was born vaginally at 25 weeks of gestation with a birth weight of 740 g, length of 36 cm, head circumference of 23 cm, and chest circumference of 21 cm. The amniotic fluid was turbid, and antenatal lung maturation therapy had not been administered. At birth, the neonate was cyanotic with a weak cry and clinical signs of respiratory distress. Apgar scores were 3 and 4 at one and five minutes, respectively.

Immediately after birth, the neonate was transferred to level III NICU, where non-invasive respiratory support with continuous positive airway pressure (CPAP) was initiated. Following chest radiography, endotracheal intubation and mechanical ventilation commenced due to deteriorating respiratory status. Porcine surfactants were administered endotracheally according to standard protocol. Blood cultures and tracheal aspirates were obtained, and empirical antibiotic therapy was initiated with ampicillin and amikacin, along with prophylactic fluconazole.

On the second day of life, due to a septic clinical presentation and abnormal laboratory parameters (white blood cell [WBC] count 99.68 × 10^9^/L, neutrophils 80.1%, lymphocytes 14.0%, platelets 478 × 10^9^/L, C-reactive protein [CRP] 4.11 mg/L), antibiotic therapy was empirically changed to meropenem (20 mg/kg/dose every 12 h) and vancomycin (10 mg/kg/dose every 18 h), preceded by appropriate microbiological sampling.

**Figure 1:**
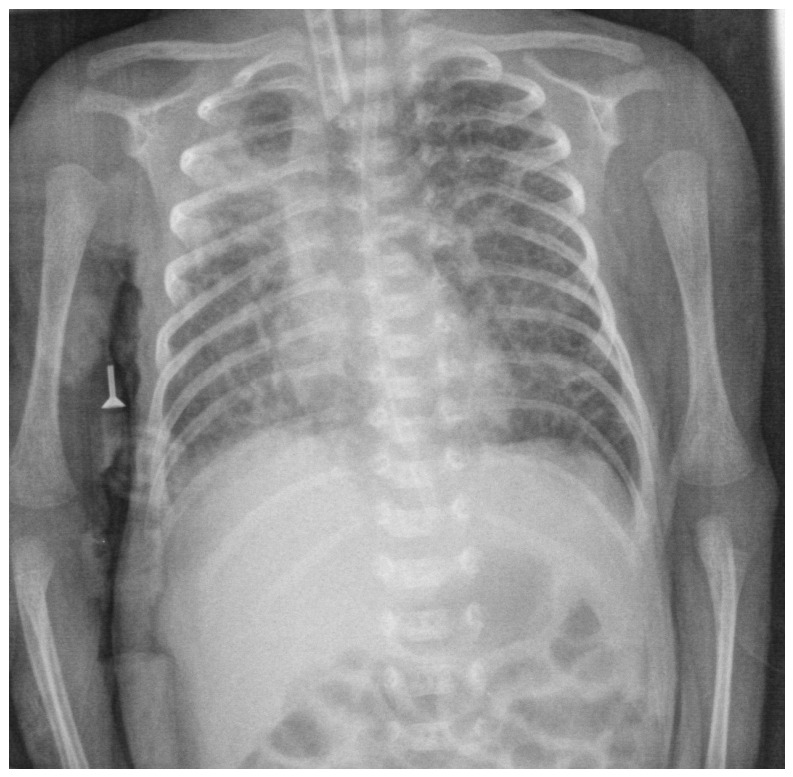
Chest X-ray showing bilateral, irregular loss of transparency of the lung parenchyma.

In response to treatment, CRP decreased to 2.6 mg/L, and the white blood cell count gradually declined during the first two weeks of life. By the end of the second week, the white blood cell count was 29.02 × 10^9^/L, neutrophils 50.9%, lymphocytes 40.9%, and platelets 315 × 10^9^/L. During the first two weeks, two unsuccessful extubation attempts were made. Due to the requirement for higher mechanical ventilation parameters, increased secretion suctioning, and radiographically confirmed pneumonia (Figure 1), a standardized endotracheal tracheal aspirate was collected on day 17 of life using a closed-suction sterile technique. The aspirate yielded *Acinetobacter* spp. susceptible to tigecycline, with intermediate susceptibility to ampicillin-sulbactam and cefepime (complete antibiogram is provided in Table 1). Antibiotic therapy was modified to cefepime (30 mg/kg/dose every 12 h) with inhaled colistin (500,000 IU every 12 h). This therapeutic choice was guided by the full susceptibility profile and by the absence of validated high-dose ampicillin-sulbactam pharmacokinetic/pharmacodynamic data for neonates of this weight class (<750 g), which precluded reliable bactericidal sulbactam exposure; inhaled colistin was added to minimize systemic toxicity while providing additional coverage against the *Acinetobacter* isolate. The chest radiograph on day 17 showed new bilateral infiltrates with consolidation (Figure 1), fulfilling CDC neonatal VAP criteria together with clinical and laboratory parameters described above.

On day 24 of life, due to clinical deterioration, markedly elevated inflammatory markers (interleukin-6 [IL-6]: 677.4 pg/mL), and pathological radiographic findings, cefepime was discontinued and tigecycline was initiated according to the antibiogram (1.2 mg/kg/dose every 12 h) for the following two weeks. Repeated tracheal aspirates isolated XDR strains of *Acinetobacter calcoaceticus-baumannii* complex and *Pseudomonas aeruginosa*. On day 38, the antibiotic regimen was modified to fosfomycin (50 mg/kg/dose every 12 h) and ceftazidime–avibactam (25 mg/kg/dose every 8 h). Due to further deterioration of gas exchange and worsening radiographic findings, high-frequency ventilation was initiated with a fraction of inspired oxygen (FiO_2_) of 0.85. The XDR *Acinetobacter calcoaceticus-baumannii* complex persisted in subsequent tracheal aspirates.

On day 43 of life, following a recommendation by the clinical pharmacologist, and after obtaining written informed parental consent given the off-label use of the drug in the neonatal age group, sulbactam–durlobactam was initiated (25 mg/kg/dose every 6 h—this amount refers to the sulbactam component alone) in combination with continued ceftazidime–avibactam. During the treatment course, renal function tests (urea and creatinine) and liver function tests Aspartate Aminotransferase (AST) and Alanine Aminotransferase (ALT) were monitored regularly, with no values outside the reference range. After 12 days of this regimen, the neonate was successfully extubated. In the subsequent period, the infant intermittently required nasal CPAP and supplemental oxygen via nasal cannula. The neonate was discharged from the neonatology ward at 42 weeks of corrected gestational age with a diagnosis of mild bronchopulmonary dysplasia (BPD), with a discharge weight of 3540 g (Table 2).

## 3. Discussion

In this case of a preterm neonate with severe pneumonia and systemic infection, initial empirical therapy consisted of a combination of vancomycin and meropenem to provide broad-spectrum coverage against potential causative pathogens, including both Gram-positive and Gram-negative bacteria [12,13,14,15]. However, this initial therapy proved ineffective in our patient.

Tigecycline, a broad-spectrum glycylcycline antibiotic active against multidrug-resistant (MDR) and XDR pathogens, has shown good clinical efficacy and acceptable tolerability in severe pediatric infections, particularly those caused by *Acinetobacter baumannii* [16,17]. Despite tigecycline administration, our patient achieved only partial clinical improvement, highlighting the complexity of managing multidrug-resistant infections in preterm neonates and the need for further antibiotic modification and intensive supportive care.

Given the failure of preceding therapies, the introduction of ceftazidime–avibactam (CAZ-AVI) in our preterm neonate proved to be a critical strategy for infection control. Although still limited in its study in neonates, this antibiotic combination enables effective targeting of multidrug-resistant Gram-negative bacteria, including extended-spectrum β-lactamases (ESBL)-producing and Klebsiella pneumoniae carbapenemase (KPC-)positive Klebsiella pneumoniae strains [18,19]. Combination with fosfomycin or amikacin, based on previous studies, may further reduce the risk of secondary infections and the emergence of resistance [20]. A randomized controlled trial involving 120 neonates (aged ≤ 28 days) demonstrated that fosfomycin can be safely used as an adjunct to standard therapy for neonatal sepsis, without significant effects on serum sodium levels or gastrointestinal adverse events, with proposed dosing effective across different neonatal age and weight groups [21].

Following the limited clinical response to prior reserve antibiotics (vancomycin, meropenem, tigecycline, and CAZ-AVI combined with fosfomycin), the therapeutic regimen was modified by incorporating sulbactam–durlobactam (SUL-DUR). Sulbactam is a beta-lactam antibiotic with intrinsic bactericidal activity against *Acinetobacter* spp. through inhibition of penicillin-binding proteins (PBPs), particularly PBP1 and PBP3. Durlobactam is a diazabicyclooctane (DBO) non-beta-lactam beta-lactamase inhibitor that potently inhibits class A (including KPC), class C (AmpC), and class D (OXA-type) serine beta-lactamases, thereby restoring sulbactam activity against isolates that would otherwise be resistant. Major resistance mechanisms discussed include: (1) overexpression of OXA-23 or OXA-51-type carbapenemases with reduced susceptibility to durlobactam; (2) metallo-beta-lactamases (class B, e.g., NDM, VIM) which are not inhibited by durlobactam; (3) loss of outer membrane porins (e.g., OmpA, CarO) reducing drug penetration; and (4) upregulation of efflux pumps (e.g., AdeABC, AdeFGH) [22]. Although this combination is approved for adult patients with hospital-acquired and ventilator-associated pneumonia caused by the *Acinetobacter baumannii*-calcoaceticus complex, its use in children and neonates remains rare, with only isolated clinical reports of SUL-DUR administration in infants with severe sepsis caused by carbapenem-resistant *Acinetobacter*, resulting in clinical improvement without serious adverse effects. These findings suggest that sulbactam–durlobactam may represent a useful therapeutic option in severe MDR/XDR infections when conventional and other reserve therapies have proven inadequate, but further clinical studies are needed to define the optimal dosing regimen and safety profile in the neonatal population [23].

The decision to combine sulbactam–durlobactam with ceftazidime–avibactam, rather than with meropenem or imipenem/cilastatin as recommended in current adult guidelines, was driven by several patient-specific factors. Meropenem had been part of multiple prior regimens without achieving microbiological eradication, making re-exposure unlikely to provide meaningful synergistic benefit against an XDR strain selected under prolonged carbapenem pressure. Additionally, the concurrent XDR Pseudomonas aeruginosa infection favored retention of CAZ-AVI, which retains activity against many carbapenem-resistant Pseudomonas isolates and thus provided rational dual-pathogen coverage that a carbapenem could not replicate. CAZ-AVI had also already been initiated on day 38 with partial clinical stabilization, and its continuation avoided additional nephrotoxic exposure in an extremely preterm neonate with immature renal function. It should also be noted that the recommendation for SUL-DUR combined with a carbapenem derives exclusively from adult trial data, with no neonatal evidence to confirm that this strategy is either necessary or optimal in a population with substantially different pharmacokinetics. Recent consensus-based pediatric dosing recommendations for novel β-lactam agents in antimicrobial-resistant Gram-negative infections emphasize a structured extrapolation of pharmacokinetic and pharmacodynamic principles in the absence of direct pediatric data. In this context, Lockowitz et al. provide a comprehensive expert-derived framework for dosing of select β-lactam antibiotics in neonates, infants, children, and adolescents, highlighting the reliance on available pharmacokinetic data, adult studies, and established pediatric experience with related agents [24]. Specifically, for sulbactam–durlobactam, pediatric pharmacokinetic data are currently unavailable, and dosing strategies are therefore inferred from the well-established use of ampicillin–sulbactam in children and general β-lactam PK/PD behavior in pediatric populations. Although adult data demonstrate favorable pharmacodynamic target attainment and clinical efficacy in carbapenem-resistant *Acinetobacter baumannii* infections, pediatric use remains extrapolative and requires careful consideration of PK/PD principles until dedicated pediatric studies become available [24]. A Phase 1b clinical study has been initiated to investigate the pharmacokinetics and safety of SUL-DUR from birth to 18 years of age in patients with *Acinetobacter baumannii*-calcoaceticus complex (ABC) infections, including term and preterm neonates older than 7 days. These initiatives indicate growing interest in SUL-DUR as a potential therapeutic option in severe MDR/XDR infections in children, but additional clinical studies are needed to establish optimal dosing regimens and adverse effect profiles [25].

The delayed introduction of SUL-DUR on day 43 reflected the procedural requirements of responsible off-label prescribing in neonates—including clinical pharmacologist consultation and written informed parental consent—rather than failure to recognize earlier the need for escalation. This highlights a broader systemic challenge: the regulatory steps essential for patient protection in off-label neonatal prescribing can introduce clinically significant delays in the context of life-threatening XDR infections, underscoring the need for streamlined compassionate use pathways in neonatal intensive care settings.

Several limitations of this case report must be acknowledged. First, as an inherent limitation of single-patient case reports, no causal relationship between sulbactam–durlobactam administration and clinical improvement can be established with certainty; a delayed bactericidal effect of prior antibiotic courses or spontaneous resolution of colonization cannot be entirely excluded as contributing factors to the observed outcome. Second, the microbiological diagnosis of VAP was based on endotracheal tracheal aspirate cultures rather than bronchoalveolar lavage, which limits the ability to definitively distinguish true lower respiratory tract infection from airway colonization. Third, molecular characterization of resistance genes (e.g., metallo-beta-lactamase PCR, whole-genome sequencing) was not performed due to the unavailability of this diagnostic infrastructure at our institution, thereby precluding definitive characterization of the resistance mechanisms operative in the XDR isolates. Fourth, several antibiotic choices in this case—specifically the use of cefepime combined with inhaled colistin, tigecycline without concurrent high-dose ampicillin-sulbactam, and sulbactam–durlobactam without a carbapenem partner—deviated from current international evidence-based recommendations for the treatment of carbapenem-resistant *Acinetobacter baumannii*-calcoaceticus complex infections. Tigecycline monotherapy for severe VAP remains controversial, especially in an extremely premature neonate. These decisions were driven by the extreme prematurity of the patient (birth weight 740 g, 25 weeks of gestation), the absence of validated pharmacokinetic/pharmacodynamic data supporting adequate sulbactam exposure at standard doses in neonates of this weight class, the unavailability of real-time sulbactam therapeutic drug monitoring, established carbapenem resistance precluding the addition of meropenem to the sulbactam–durlobactam regimen, and real-time clinical judgment under resource constraints. These deviations are recognized as limitations and should be considered when interpreting the clinical response and the apparent efficacy of the final antibiotic regimen. Fifth, long-term safety and neurodevelopmental follow-up data beyond the hospital discharge at 42 weeks of corrected gestational age are not yet available, as structured follow-up assessments at 3 and 6 months of corrected age are planned but have not been completed at the time of manuscript submission. Sixth, in this case, a delayed response to prior antibiotics could not be excluded with certainty. Finally, the very limited published experience with sulbactam–durlobactam in neonates means that the dosing regimen used in this case (25 mg/kg/dose every 6 h) was empirically derived and cannot be considered validated; prospective pharmacokinetic studies in this population are urgently needed.

## 4. Conclusions

This case underscores the urgent need for evidence-based guidance on the use of novel reserve antibiotics in the neonatal population. The off-label administration of sulbactam–durlobactam was undertaken as a last-resort measure in a life-threatening situation, with written parental consent and pharmacological guidance. Tolerability was satisfactory, as evidenced by clinical and laboratory monitoring throughout the treatment course. Our report adds to the very limited body of literature on SUL-DUR in neonates and supports the initiation of controlled pharmacokinetic and safety studies in this vulnerable age group.

## Figures and Tables

**Table 1:** Susceptibility profile of *Acinetobacter* and *Pseudomonas aeruginosa* isolates.

Antibiotic	*Acinetobacter* Species	*Acinetobacter calcoaceticus-baumannii* Complex	*Pseudomonas aeruginosa*
Ampicillin–sulbactam	I	R	Not tested
Piperacillin–tazobactam	R	R	R
Cefotaxime	R	R	R
Cefepime	S	R	R
Ceftazidime	R	R	R
Ceftriaxone	R	R	R
Imipenem–cilastatin	R	R	R
Imipenem–cilastatin–relebactam	R	R	R
Meropenem	R	R	R
Amikacin	R	R	R
Gentamycin	R	R	R
Tobramycin	Not tested	Not tested	I
Ciprofloxacin	R	R	R
Levofloxacin	R	R	R
Colistine	Not tested	Not tested	S
Tigecycline	S	S	Inherently resistant, not tested

R—resistant; S—sensitive; I—intermediate. Note: Interpretation of microbiologic data remains limited due to the unavailability of minimal inhibitory concentrations (MICs).

**Table 2:** Management timeline of the patient.

Day of Hospitalization	Respiratory Support (Ventilation Mode/Settings)	Microbiological Results	Inflammatory Markers (CRP/IL-6/WBC)	Antibiotic Therapy Changes
Day 0	Mechanical ventilation	Blood cultures and tracheal aspirate obtained		Ampicillin + amikacin + fluconazole prophylaxis
Day 2	Mechanical ventilation		WBC 99.68 × 10^9^/L; neutrophils 80.1%; CRP 4.11 mg/L	Switched to meropenem + vancomycin
Days 1–14	Mechanical ventilation; 2 failed extubation attempts		CRP ↓ to 2.6 mg/L; WBC ↓ to 29.02 × 10^9^/L	Continued meropenem + vancomycin
Day 17	Mechanical ventilation (increased settings)	*Acinetobacter* spp.	IL-6: 20.2 pg/mL; WBC ↓ to 24.11 × 10^9^/L	Switched to cefepime + inhaled colistin
Day 24	Mechanical ventilation; worsening gas exchange	XDR *Acinetobacter calcoaceticus-baumannii* complex + *Pseudomonas aeruginosa*	IL-6: 677.4 pg/mL	Cefepime discontinued → tigecycline
Day 38	High-frequency ventilation (FiO_2_ 0.85)	Persistent XDR *Acinetobacter calcoaceticus-baumannii* complex	WBC ↓ to 13.54 × 10^9^/L	Fosfomycin + ceftazidime–avibactam
Day 43	High-frequency ventilation	Persistent XDR *Acinetobacter calcoaceticus-baumannii* complex		Sulbactam–durlobactam + ceftazidime–avibactam
~Day 55	Intermittent CPAP/nasal cannula oxygen	Clearance of infection (no new isolate reported)		Antibiotics discontinued
Day 119: Discharge	Breathing room air	No new positive cultures	Stable inflammatory markers	No ongoing antibiotic therapy

Note: ↓: decrease; →: treatment switch; ~: approximately.

## Data Availability

The data presented in this study are available upon request from the corresponding author due to the GDPR protocols of the hospitals involved in the treatment of the reported patient.

## Abbreviations

The following abbreviations are used in this manuscript:

VAP	Ventilator-associated pneumonia
NICUs	Neonatal intensive care units
MDR	Multidrug-resistant
XDR	Extensively drug-resistant
CDC	Centers for Disease Control and Prevention
MRSA	Methicillin-resistant Staphylococcus aureus
CPAP	Continuous positive airway pressure
CRP	C-reactive protein
FiO_2_	Fraction of inspired oxygen
BPD	Bronchopulmonary dysplasia
PK/PD	Pharmacokinetic/pharmacodynamic
CAZ-AVI	Ceftazidime–avibactam
SUL-DUR	Sulbactam–durlobactam
ABC	*Acinetobacter baumannii*-calcoaceticus complex
